# Supplementary material for: Biodiversity and Bioprospecting of Fungal Endophytes from the Antarctic Plant Colobanthus quitensis
Source: J Fungi (Basel). 2022 Sep 19;8(9):979. doi: 10.3390/jof8090979 (PMC9504944; doi:10.3390/jof8090979)
Supplement: Supplementary file 1 [file jof-08-00979-s001.zip › jof-1876388-supplementary.pdf]

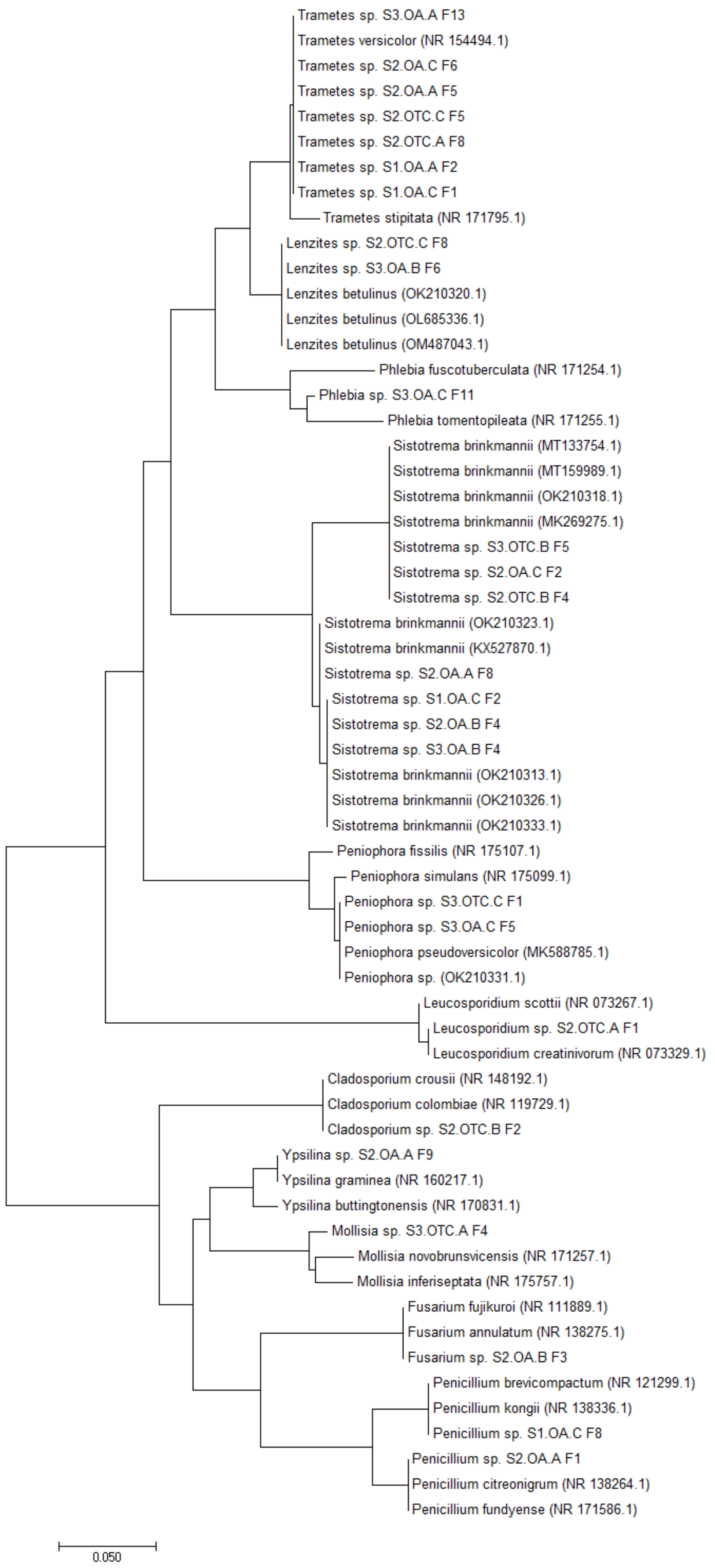

Figure S1. Phylogenetic tree of representative endophytic fungi of *Colobanthus quitensis*. The analysis was conducted with MEGA version 6.0 using the two best BLAST hits of ITS gene sequences at NCBI. The scale bar represents the number of substitutions per site.

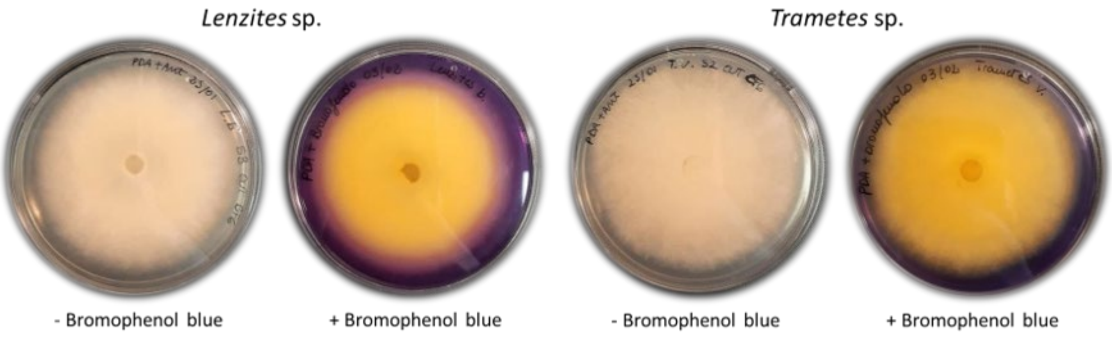

Figure S2. Representative images showing *Trametes* sp. S2.OA.C_F6 and *Lenzites* sp. S3.OA.B_F6 isolates grown for 7 days on PDA solid medium (pH 5.6) in the presence (+) or absence (-) of bromophenol blue, as a pH indicator (yellow, pH < 3.5; blue, pH > 4.6).

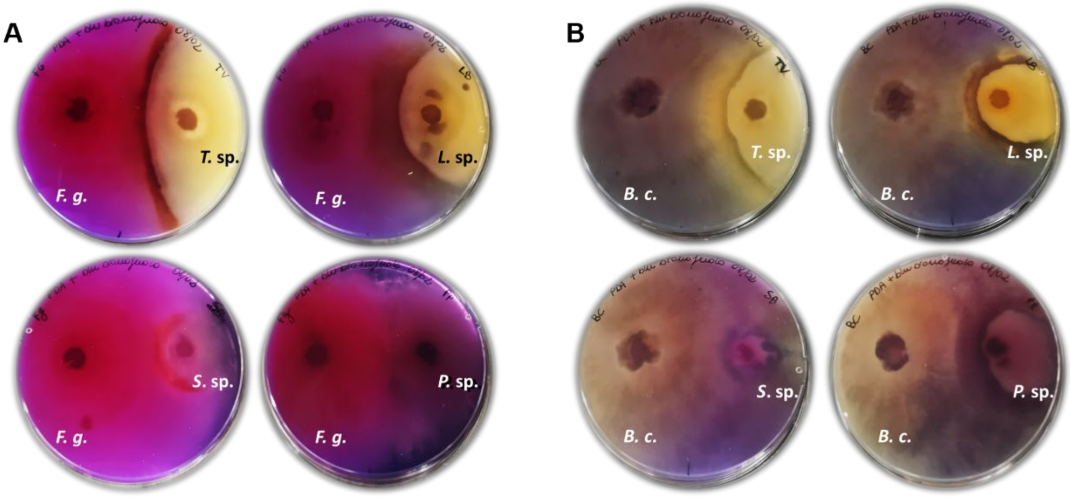

Figure S3. Co-cultivation of *Fusarium graminearum* (A) and *Botrytis cinerea* (B) (on the left of the plates) and Antarctic fungal isolates on PDA solid medium at 10 dpi in the presence of bromophenol blue, as a pH indicator. *F. g.*: *Fusarium graminearum*; *B. c.*: *Botrytis cinerea*; *T.* sp.: *Trametes* sp. S2.OA.C_F6; *L.* sp.: *Lenzites* sp. S3.OA.B_F6; *S.* sp.: *Sistotrema* sp. S1.OA.C_F2; *P.* sp.: *Peniophora* sp. S3.OTC.C_F1.

Table S1. Taxonomic annotation of representative endophytic fungi of *Colobanthus quitensis*. Fungi isolated from each *C. quitensis* biological replicate (named A, B and C) collected in open areas (OA) of the Antarctic site 1 (S1), site 2 (S2) and site 3 (S3) or inside open top chambers (OTC), which were present only in S2 and S3, were taxonomically annotated by the amplification of the fungal ITS region. Accession numbers of NCBI (http://www.ncbi.nlm.nih.gov/sra) are indicated for each fungal isolate.

**Representative fungal isolates**		
**Isolate code**	**Taxonomic annotation**	**NCBI accession number**
S1.OA.A_F2	*Trametes* sp.	OK210311
S1.OA.C_F1	*Trametes* sp.	OK210312
S1.OA.C_F2	*Sistotrema* sp.	OK210313
S1.OA.C_F8	*Penicillium* sp.	OK210314
S2.OTC.A_F1	*Leucosporidium* sp.	OK210315
S2.OTC.A_F8	*Trametes* sp.	OK210316
S2.OTC.B_F2	*Cladosporium* sp.	OK210317
S2.OTC.B_F4	*Sistotrema* sp.	OK210318
S2.OTC.C_F5	*Trametes* sp.	OK210319
S2.OTC.C_F8	*Lenzites* sp.	OK210320
S2.OA.A_F1	*Penicillium* sp.	OK210321
S2.OA.A_F5	*Trametes* sp.	OK210322
S2.OA.A_F8	*Sistotrema* sp.	OK210323
S2.OA.A_F9	*Ypsilina* sp.	OK210324
S2.OA.B_F3	*Fusarium* sp.	OK210325
S2.OA.B_F4	*Sistotrema* sp.	OK210326
S2.OA.C_F2	*Sistotrema* sp.	OK210327
S2.OA.C_F6	*Trametes* sp.	OK210328
S3.OTC.A_F4	*Mollisia* sp.	OK210329
S3.OTC.B_F5	*Sistotrema* sp.	OK210330
S3.OTC.C_F1	*Peniophora* sp.	OK210331
S3.OA.A_F13	*Trametes* sp.	OK210332
S3.OA.B_F4	*Sistotrema* sp.	OK210333
S3.OA.B_F6	*Lenzites* sp.	OM487043
S3.OA.C_F5	*Peniophora* sp.	OK210334
S3.OA.C_F11	*Phlebia* sp.	OK210335

Table S2. Highest cellulolytic and amylolytic activities of selected Antarctic fungal isolates detected *in vitro* on carboxymethylcellulose (CMC) or soluble starch. Optimal pH values are reported within brackets. Experiments were conducted in triplicate and standard deviation (SD) is reported.

**Fungal isolate**	**Cellulolytic activity on CMC (nKat mL^{-1})**	**Amylolytic activity on starch (nKat mL^{-1})**
Trametes sp. S1.OA.A_F2	0.463 ± 0.04 (pH 7)	0.199 ± 0.03 (pH 7)
Trametes sp. S2.OTC.C_F5	0.494 ± 0.05 (pH 4)	0.155 ± 0.02 (pH 7)
Trametes sp. S2.OA.A_F5	0.201 ± 0.03 (pH 4)	0.513 ± 0.04 (pH 7)
Lenzites sp. S3.OA.B_F6	0.182 ± 0.02 (pH 5)	4.676 ± 0.35 (pH 5)

Table S3. Total phenolic content (TPC) and total flavonoid content (TFC) of aqueous (Aq) or methanol (Met) extract of *Trametes* sp. S2.OA.C_F6, *Lenzites* sp. S3.OA.B_F6 and *Sistotrema* sp. S1.OA.C_F2. Values were reported as mean ± SD of triplicates.

Fungal isolate	TPC (mg GAE g^{-1} d.w.)	TFC (mg QE g^{-1} d.w.)
Trametes sp. S2.OA.C_F6 Aq	2.667 ± 0.017	4.389 ± 0.016
Lenzites sp. S3.OA.B_F6 Aq	4.087 ± 0.050	5.516 ± 0.022
Sistotrema sp. S1.OA.C_F2 Aq	1.086 ± 0.010	1.626 ± 0.009
Trametes sp. S2.OA.C_F6 Met	6.058 ± 0.056	2.437 ± 0.024
Lenzites sp. S3.OA.B_F6 Met	3.803 ± 0.031	1.964 ± 0.001
Sistotrema sp. S1.OA.C_F2 Met	1.770 ± 0.013	1.310 ± 0.014
